# MiRNA-186 as a Biomarker of Disease Exacerbation in Rheumatoid Arthritis: Insights from Clinical Data and Molecular Marker Analysis

**DOI:** 10.3390/ijms26168039

**Published:** 2025-08-20

**Authors:** Marek Cieśla, Dorota Darmochwał-Kolarz, Hubert Kubis, Bogdan Kolarz

**Affiliations:** 1 Laboratory of Diagnostic and Clinical Epigenetics, Faculty of Medicine, University of Rzeszow, 35-310 Rzeszow, Poland; 2 Department of Obstetrics and Gynecology, Faculty of Medicine, University of Rzeszow, 35-310 Rzeszow, Poland; 3 Department of Rheumatology, Faculty of Medicine, University of Rzeszow, 35-310 Rzeszow, Poland

**Keywords:** DAS28, disease activity, microRNA, miR-186, noncoding RNA, rheumatoid arthritis

## Abstract

Rheumatoid arthritis (RA) is a chronic, systemic autoimmune disease characterized by inflammation of the synovial tissue, leading to joint destruction, pain, stiffness, and progressive impairment of motor functions. Despite significant advances in diagnosis and treatment, RA remains a major clinical and social challenge, negatively impacting patients’ quality of life. The aim of this study was to assess the relationship between the expression of selected microRNAs (miRNAs) and the activity of the disease. A total of 46 RA patients and 20 healthy controls (HCs) were enrolled in the study. A quantitative real-time polymerase chain reaction was used to evaluate the expression of miRNAs in whole blood. MiRNA-186 exhibited decreased concentrations in RA patients compared to HCs (*p* = 0.03). Patients with an active form of the disease (DAS28 > 3.2) exhibited lower expression of miRNA-186 than HCs (*p* = 0.04). Additionally, ACPA-negative patients also demonstrated reduced miRNA-186 expression compared to controls. AUC analysis confirmed that the combination of miRNA-186, the erythrocyte sedimentation rate (ESR), and Visual Analog Scale—Patient Global Assessment (VAS PGA) may be effective in identifying RA exacerbation. The combination of classical laboratory markers, clinical data, and molecular markers enhances the ability to assess RA exacerbation. MiRNA-186 may be considered a potential marker of disease activity in RA.

## 1. Introduction

Noncoding RNA (ncRNA) is a class of molecules that do not encode proteins but are involved in the regulation of gene expression and thereby influence numerous biological processes. From a historical perspective, the central dogma of molecular biology emphasized the role of messenger RNAs in protein synthesis, overshadowing the potential biological functions of noncoding RNAs. At that time, ncRNAs were considered molecular junk or background noise in the cell [1]. The role of ribosomal RNA and transfer RNA in gene expression was already established in the 1950s, but it was the discovery of microRNA (miRNA) in the early 2000s that accelerated research into the functions of other classes of noncoding RNAs [2]. It is now known that transcription may involve more than four-fifths of the genomic DNA, while only approximately 1.5% of the transcriptome corresponds to protein-coding sequences, indicating that RNA molecules without coding potential are abundant. Since then, growing evidence has supported their role in gene expression regulation, including transcriptional, post-transcriptional, and epigenetic mechanisms. NcRNAs are involved in various cellular processes such as messenger RNA degradation, alternative splicing, chromatin remodeling, and the maintenance of genomic stability [1,3].

NcRNAs can be divided into two classes depending on their length: long ncRNAs, such as long noncoding RNAs (lncRNAs) and circular RNAs, as well as short molecules, including miRNAs, small nuclear RNAs, and small nucleolar RNAs [3,4]. MiRNAs are endogenous, small, single-stranded ncRNAs approximately 18–25 nucleotides in length. The biogenesis of miRNAs involves transcription of their sequences by RNA polymerase II, resulting in the formation of primary miRNAs (pri-miRNAs), which adopt a hairpin structure. The maturation of pri-miRNAs is mediated by two RNase III-type enzymes, Drosha and Dicer, which sequentially cleave the hairpin arms [5]. Cleavage by Drosha produces precursor miRNAs (pre-miRNAs), which are transported by exportin-5 to the cytoplasm for further processing by the Dicer protein [6,7,8]. The mature miRNA is then loaded onto the Argonaute (Ago) protein to form the RNA-induced silencing complex (RISC). In addition to regulating gene expression, miRNAs are involved in several fundamental biological processes, including cell proliferation, apoptosis, and immune system regulation [9,10].

Rheumatoid arthritis (RA) is a chronic autoimmune disorder that causes systemic inflammation throughout the body. One of the most common symptoms of the disease is symmetrical joint pain, swelling, and stiffness. In the advanced stages of the disease, there is an overproduction of pro-inflammatory cytokines, such as tumor necrosis factor (TNF) and interleukin-6 (IL-6), which damage synovial cells and contribute to cartilage and bone destruction [11]. RA is a multifactorial disease in which environmental, genetic, and epigenetic factors contribute to its development. Among epigenetic factors, the main regulatory mechanisms are considered to be DNA methylation, histone protein modifications, and the influence of ncRNAs on gene expression regulation [12,13].

MiRNAs in RA are extensively studied in serum, plasma, whole blood, peripheral blood mononuclear cells (PBMCs), and various tissues [14]. Many of these molecules contribute to the overproduction of pro-inflammatory cytokines and the activation of leukocytes, both of which play a role in the pathogenesis and progression of RA [15,16]. Changes in miRNAs expression may serve as potential biomarkers for disease diagnosis, exacerbation, or response to treatment. This is because miRNAs are tissue-specific, easily measurable, and cost-effective, particularly when the polymerase chain reaction (PCR) is used as a method with high specificity and sensitivity [14,17,18].

The aim of this investigation was to evaluate the association between the expression level of selected miRNAs in PBMCs, whose quantitative alterations may be linked to RA severity. Based on a review of the literature, we selected five miRNAs, miRNA 186-5p (miRNA-186), miRNA 654-3p (miRNA-654), miRNA 425-5p (miRNA-425), miRNA 22-3p (miRNA-22), and miRNA106b-5b (miRNA-106b), which have been previously associated with RA [19,20,21,22].

## 2. Results

### 2.1. MicroRNA Expression and Clinical Data

Of the five miRNA molecules evaluated, only miRNA-186 exhibited an approximately 73% decrease in expression in patients with RA compared to healthy controls (HCs). Details are presented in Table 1.

Subsequently, patients with RA were divided into groups based on disease activity, according to the disease activity score for 28 joints (DAS28), the Simplified Disease Activity Index (SDAI), and the Clinical Disease Activity Index (CDAI). Based on the given activity scales, the patients were categorized into two subgroups: active disease and inactive disease. The active disease group included patients with moderate to high disease activity (DAS28 > 3.2, n = 30), while the inactive disease group included patients in remission or with low disease activity (DAS28 ≤ 3.2, n = 16). Details are presented in Table 2.

For the SDAI scale, the active form of the disease was represented by 32 patients with RA (SDAI > 11), while the inactive form included 14 patients (SDAI ≤ 11; including those with low disease activity and remission). Details are presented in Table 3.

For the CDAI scale, the active form of the disease was represented by 32 patients with RA (CDAI > 11), while the inactive form included 14 patients with RA (CDAI ≤ 11; including those with low disease activity and remission). Details are presented in Table 4.

**Figure 1:**
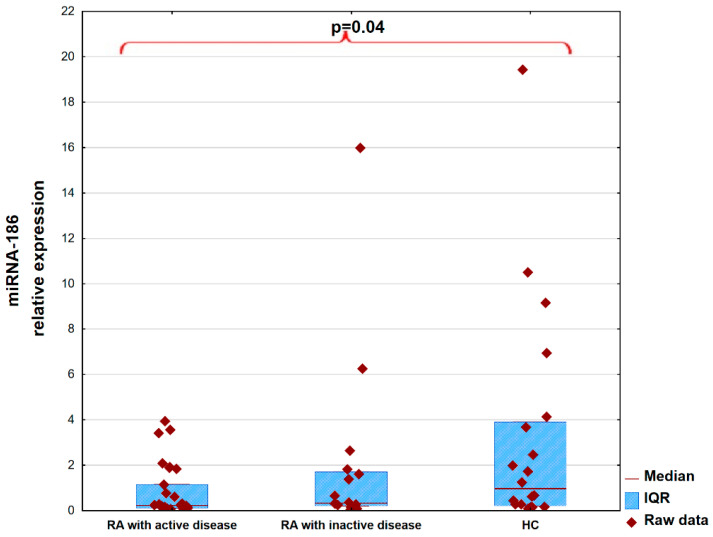
MicroRNA-186 expression level in patients with rheumatoid arthritis divided according to disease activity and in the control group.

Post hoc analysis showed that the expression level of miRNA-186 was decreased in RA patients with active disease compared to the HC group, across all activity measures, including the DAS28, SDAI, and CDAI (*p* = 0.04, *p* = 0.04, and *p* = 0.04, respectively). Details regarding disease activity based on the DAS28 are presented in Figure 1.

**Figure 2:**
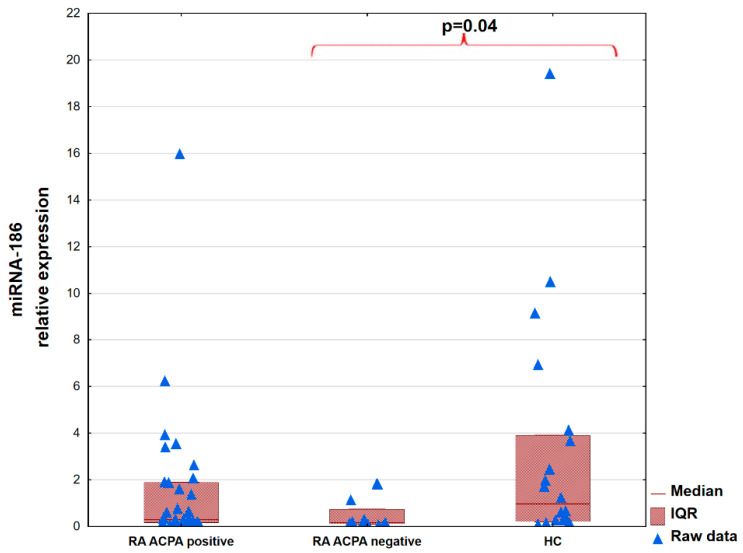
Level of expression of microRNA-186 in patients with RA divided according to the presence of ACPAs and in the control group.

There were no significant differences among the patients divided according to the presence of rheumatoid factor (RF; seropositive vs. seronegative) and the HC group. Details are presented in Supplementary Table S2. Patients with RA seronegative for anti-citrullinated protein antibodies (ACPAs; median; [IQR]: 0.16 [0.12–0.73] had decreased expression of miRNA-186 in comparison to HCs: 0.96 [0.22–3.91]; *p* = 0.04. Details are presented in Figure 2 and Supplementary Table S3.

The correlation analysis between miRNAs and clinical variables—DAS28, CDAI, SDAI, erythrocyte sedimentation rate (ESR), number of swollen joints (SJN), number of tender joints (TJN), RF, ACPAs—was conducted in the RA group. Only miRNA-654 showed a negative correlation with the ESR (rs = −0.29). Details on the strength and direction of individual correlations are included in Supplementary Table S4.

### 2.2. MicroRNA Expression and Diagnostic Potential

**Figure 3:**
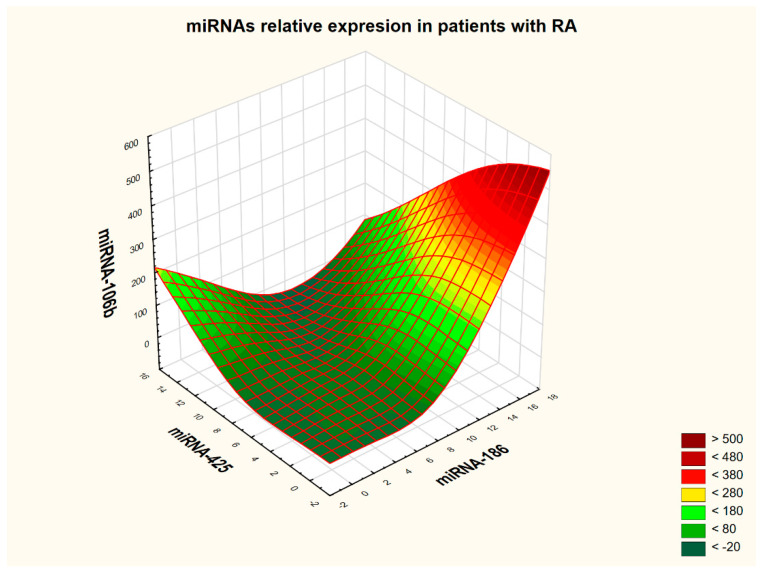
Relative expression of microRNAs in patients with rheumatoid arthritis. The 3D plots were generated using distance-weighted least-squares smoothing, allowing for localized surface fitting based on the proximity of data points. Each axis (X, Y, and Z) represents an independent variable, enabling the simultaneous analysis of three distinct parameters. The color scale ranges from dark green to red, indicating the intensity of the smoothed values—where dark green corresponds to lower values and red to higher ones. This visualization approach facilitates the identification of complex relationships and local patterns within multi-dimensional data.

**Figure 4:**
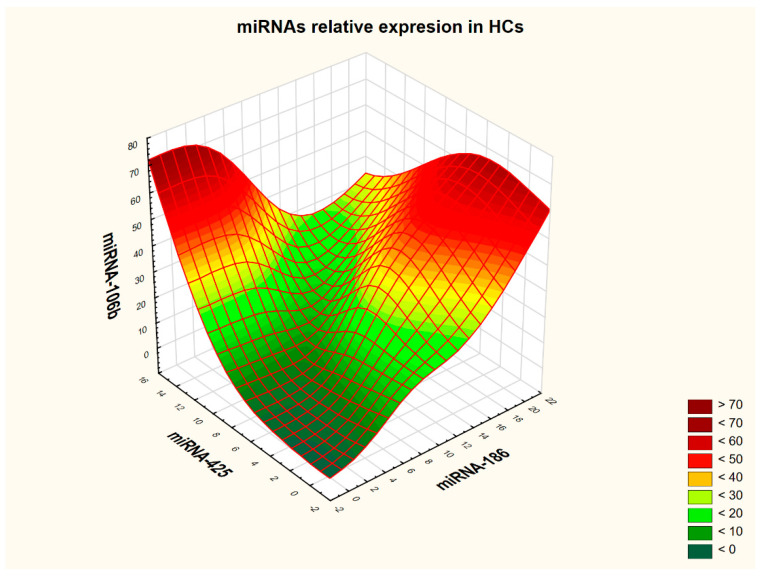
Relative expression of microRNAs in healthy controls. For interpretation of the 3D plot, please refer to the description provided with Figure 3.

Correlation analysis between individual miRNAs was performed in the RA group and in the HC group, respectively. Details are presented in Table 5 and Table 6. The strongest correlations (≥0.9) were observed among three miRNAs, miRNA 186, miRNA-425, and miRNA-106b, in both the RA patient group and in the control group. The interdependence of expression among these three molecular targets is visualized in Figure 3 and Figure 4. To generate three-dimensional graphs, the distance-weighted least-squares smoothing method was used.

Analysis of the 3D graphs suggests that the two study groups exhibit distinct expression profiles of the three analyzed miRNAs when their mutual interactions are considered. In the control group, HCs demonstrated the following molecular profile: high miRNA-106b expression, high miRNA-186 expression, and moderate miRNA-425 expression. In contrast, the RA patient group was characterized by moderate expression of miRNA-106b, high expression of miRNA-186, and low expression of miRNA-425. To verify this observation, the receiver operating characteristic (ROC) analysis was performed—first for serological markers such as ACPAs and RF, and then for the individual miRNAs. Subsequently, a combined analysis of antibody and miRNA expression was conducted. ROC analysis confirmed previous findings, indicating that only miRNA-186 was capable of distinguishing RA patients from HCs; however, the area under the curve (AUC) value, although statistically significant, was lower than that of classical serological markers. Details are presented in Table 7.

The application of the logistic regression model suggested that the combination of ACPAs and at least one miRNA—excluding miRNA-22—may enhance the diagnostic value of these markers. Notably, the combination of ACPAs and miRNA-654 (AUC = 0.941) showed no relevant difference compared to the combination of ACPAs with all tested miRNAs (AUC = 0.949). Details are presented in Table 8.

### 2.3. MicroRNAs as Markers of Disease Activity

**Figure 5:**
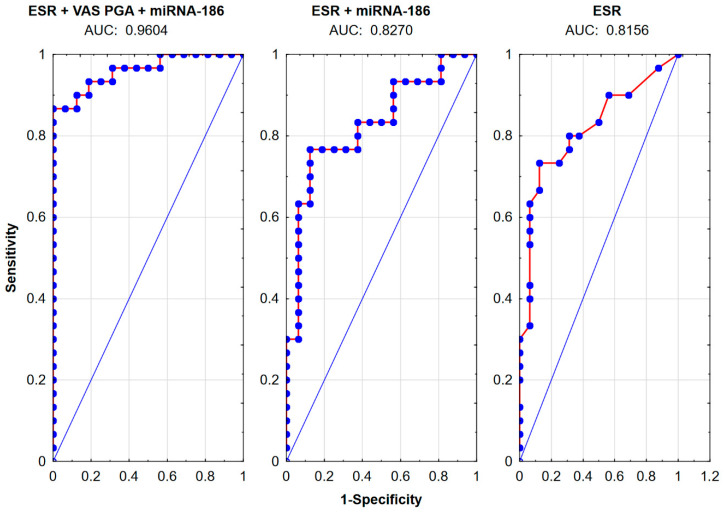
Receiver operating characteristic (ROC) analysis applied to the evaluation of disease activity markers in rheumatoid arthritis. Abbreviations AUC, area under the curve; ESR, rate of erythrocyte sedimentation; miRNA, microRNA; VAS PGA, Visual Analog Scale–Patients Global Assessment.

The logistic regression model was used to estimate the ability to assess disease activity in patients with RA, categorized into active and inactive disease groups according to the DAS28 scale. A comparison of the potential utility of the studied miRNAs as additional variables for identifying disease exacerbation is presented in Table 9 and Figure 5.

### 2.4. Interaction Between MiRNA-186 and Its Potential Target Genes

The in silico analysis revealed several dozen potential genes that may be regulated by miRNA-186. The highest number of predicted interactions—13 transcripts—were identified within the coding sequence (CDS) regions of target genes. Additionally, three genes showed interactions within the 3′ untranslated region (3′ UTR) and one gene within the 5′ untranslated region (5′ UTR). Detailed data are provided in Supplementary Table S5.

## 3. Discussion

In this study, we demonstrated the novel finding that miRNA-186 may serve as a potential molecular marker associated with disease activity exacerbation and may be useful for diagnosing patients with rheumatoid arthritis in whom the ACPA has not been detected.

Previous studies have reported that miRNA-186 may act as a pro-inflammatory molecule. The study conducted by Singh et al. [23] showed upregulation of miRNA-186 in systemic lupus erythematosus (SLE) and a positive correlation with disease activity. Another study [24] indicated that miRNA-186 is associated with the development of inflammation via interleukin-1-beta (IL-1β) in osteoarthritis (OA). In vitro stimulation of chondrocytes with IL-1β has been shown to increase the expression of inflammatory mediators such as TNF-α and IL-6, which are also key molecules in the pathogenesis of RA. Moreover, IL-1β was found to upregulate miRNA-186-5p expression in a cell line model. The study by Akbaba et al. [25], who compared patients with severe systemic autoinflammatory diseases (SAIDs) and mild familial Mediterranean fever (FMF), demonstrated that miRNA-186-5p is associated with inflammation. However, the authors noted that patients with SAIDs exhibited reduced levels of miRNA-186 expression compared to those with FMF. One of the most important molecules linking the pathophysiology of adaptive and innate immunity is IL-1β, which plays a central role in stimulating inflammation. The study by Akbaba et al. [25] indicated that miRNA-186 is associated with inflammation-related pathways, such as the cytokine-mediated signaling pathway and the cellular response to cytokine stimulus. In our study, we did not confirm a direct correlation between the level of miRNA-186 expression and disease activity. Furthermore, the miRNA-186 level was decreased in patients with RA, and it was also lower in the group of patients with an active form of the disease compared to healthy individuals. The underlying mechanisms of SLE, RA, and OA are fundamentally distinct. RA is a systemic autoimmune disorder primarily driven by pro-inflammatory cytokines such as TNF-α and IL-6. In contrast, SLE is considered a prototypical interferonopathy, characterized by the overexpression of genes within the “interferon signature,” which significantly contributes to disease activity. Type I interferons are known to modulate the expression of numerous miRNAs, potentially explaining the observed upregulation of miRNA-186 in SLE. OA, on the other hand, is predominantly a degenerative joint disease, where inflammation—if present—is typically secondary and low-grade [26]. These divergent pathogenic landscapes may account for the differential regulation of miRNA-186 across these conditions.

The differential expression of various ncRNAs between various disease entities is not unusual. MiRNA-146a, which is considered an anti-inflammatory molecule in SLE patients, shows a decrease in PBMCs [23], while in patients with RA, it is increased in PBMCs. The study conducted by Pauley et al. [27] showed that although the level of miRNA-146a differed between RA and controls, the two main genes responsible for the regulation of TNF-α levels, interleukin-1 receptor-associated kinase 1 (IRAK1) and TNF receptor-associated factor 6 (TRAF6), did not show significant differences in expression between the groups. Other studies confirm that various ncRNAs exhibit differential expression patterns, reflecting their distinct regulatory roles. For instance, *GAS5* is downregulated in the PBMCs of patients with SLE, while its expression is elevated in RA. Notably, in SLE, *GAS5* interacts with the MAPK signaling pathway, whereas in RA, it is associated with the AMPK pathway [28]. Similar regulatory phenomena are observed with microRNAs (miRNAs). Previous studies on cell lines have shown that knockout of miR-194 results in the dysregulation of thousands of genes—approximately 2,600 downregulated and 2,400 upregulated—highlighting the complexity of miRNA-mediated gene regulation [29]. Our in silico analysis suggests a set of potential genes that may interact with miRNA-186; however, these findings should be interpreted with caution, as only functional studies can provide definitive insights into its biological role, especially in context of RA pathogenesis.

This showed that the regulation of inflammatory mediator expression occurs at multiple levels and not through a single molecule. There is no information in the literature on the expression level of miRNA-186-5p and its association with established RA; therefore, our results require confirmation. The aim of this study was not to find a direct relationship between the expression level of the tested molecule and the disease state or to conduct a functional study but to explore the possibility of using miRNAs as additional markers that may be helpful in diagnosing RA exacerbation or in identifying RA patients who are negative for serological markers such as the ACPA.

This study confirmed that the use of single molecular markers does not contribute to achieving satisfactory diagnostic value. Similar observations were noted in our previous study on lncRNAs [30] and in other studies [31,32,33]. The use of a combination of molecular markers may contribute to an increase in diagnostic value. However, even the use of a single molecular marker in combination with established serological markers, such as the ACPA, may be beneficial. Ren et al. [34] used a combination of two piwi RNAs, for which the AUC value was 0.79. Upon adding additional markers in the form of ACPAs, the AUC increased to 0.9965, while the inclusion of RF in the diagnostic algorithm resulted in an AUC of 0.9932. Interestingly, the use of C-reactive protein (CRP) alone increased the diagnostic value of the marker combination to an AUC of 0.9289. This highlights the significant potential of combining new molecular markers with classical serological markers or general indicators of inflammation. In this study, we also confirmed such a relationship. In our study, we also observed an improvement in the AUC parameter for the miRNA combination, although a similar enhancement in diagnostic value was noted for miRNA-186. Importantly, miRNA-186 also showed differential expression between ACPA-positive and ACPA-negative RA patients. Moreover, miRNA-186 showed a consistent association with all three RA disease activity scales (DAS28, CDAI, and SDAI). Although methodological differences among these scoring systems led to slight shifts in patient distribution across activity categories, the overall trend of association with disease activity remained evident. This suggests that miRNA-186 may serve as a broadly applicable marker of disease exacerbation and diagnosis; however, this finding requires further validation.

The possibility of using a combination of markers as indicators of disease activity seems particularly important. The commonly used DAS28 scale has certain limitations. The DAS28 includes only 28 joints, mainly of the upper limbs, excluding joints such as the foot and ankle, which are frequently affected in RA. This may lead to an underestimation of disease activity. Furthermore, the scale underestimates the SJN, which is a key indicator of active inflammation. Another limitation is the use of CRP as a component in calculating the DAS28 score. CRP strongly influences the DAS28-CRP value, which favors the use of agents such as IL-6 inhibitors and Janus Kinase (JAK) inhibitors, both of which significantly reduce CRP levels regardless of actual clinical improvement. In contrast, other drugs, such as abatacept or rituximab, which do not directly affect CRP, may be unfairly evaluated as less effective. The study also highlighted the importance of including a global assessment of patients (PGA), despite the controversy that may arise from the subjective nature of health status evaluations [35]. Some studies suggest the possibility of omitting the PGA as a component in remission assessments. The study by Ferreira et al. [36] indicated that remission criteria based on three components (3V), TJN, SJN, and CRP, encompass a much larger group of patients than the four-component (4V) remission criteria, which include the PGA, and are associated with a lower risk of radiological progression. Studies have shown that some patients do not meet the 4V remission criteria solely due to a high PGA, even though they do not exhibit active inflammation. In this study, we confirmed that the use of three components, TJN, SJN, and the laboratory marker ESR (instead of CRP), may effectively distinguish the active form of the disease from low activity. However, this study also demonstrates that the PGA significantly enhances the ability to differentiate disease severity. Additionally, molecular markers alone, as well as combinations of classical laboratory markers and molecular markers, are less effective. The approach presented in this research suggests the possibility of evaluating disease exacerbation using only blood-based markers and subjective patient assessments, without the need for a clinical visit. Of course, the proposed approach is not as effective as clinical evaluations of disease activity, although it offers a potential alternative. Nevertheless, the results obtained in this study should be considered preliminary and require validation in future research.

This study has some limitations. Firstly, a limitation of this study is the relatively small sample size, particularly in subgroup analyses, which may affect the statistical power and robustness of the conclusions. Moreover, the molecular profiles observed in this cohort may reflect chronic disease processes rather than early immunopathogenic events. Further functional studies are required to elucidate the biological role of miRNA-186 and its potential involvement in the pathogenesis of rheumatoid arthritis. Therefore, caution should be exercised when generalizing these results to patients in the early stages of RA. Secondly, this study regarding the use of miRNA antibodies and ACPAs as diagnostic markers is limited by the disease duration in patients with RA. Our study group had a median disease duration of approximately 12 years; therefore, the conclusions drawn should be validated in newly diagnosed patients and individuals with pre-RA, in whom the presence of ACPAs may precede the clinical symptoms of the disease by several years. Validation should also include patients with other systemic autoimmune diseases or connective tissue disorders. Notably, ACPAs appear in serum long before the onset of clinical symptoms of joint damage. Furthermore, they are stable over time, and the rate of seroconversion from ACPA-negative to ACPA-positive is very low, primarily occurring in patients who are RF-positive [37]. For this reason, it seems reasonable to consider combining this marker with molecular indicators.

The last limitation is that RA patients were treated with various classes of medications, including combination therapies. Due to the exploratory nature of this study, as well as the limited number of patients in individual treatment subgroups, this study does not allow for definitive conclusions about the influence of therapy on the expression of the analyzed miRNAs. Further research is needed to determine the relationship between miRNA-186 expression and the pharmacological treatment applied.

## 4. Materials and Methods

### 4.1. Patients

A total of 66 individuals were included in the study: 20 HCs and 46 RA patients. Patients with RA were diagnosed based on the 2010 ACR/EULAR classification criteria. The clinical variables included in the patient characteristics were obtained from medical records. Rheumatoid factor (Rheumatoid Factor IgG ELISA kit, Demeditec Diagnostics, Kiel, Germany) and ACPAs (EIA CCP IgG, TestLine Clinical Diagnostics, Brno, Czech Republic) were measured in serum using the enzyme-linked immunosorbent assay. The study was approved by the Bioethics Committee of the University of Rzeszow (protocol number 9/11/2020, approved on 19 November 2020) and conducted in accordance with the Declaration of Helsinki. All participants provided written informed consent prior to participation in this study and related procedures. The criteria for exclusion included a documented history of other autoimmune disorders (such as autoimmune thyroiditis), inflammatory conditions, overlap syndromes involving connective tissue diseases, and severe comorbidities (including active infections, malignancies, advanced heart failure, or end-stage renal disease), as well as any serious illness occurring during hospitalization. The control group comprised individuals admitted for conditions unrelated to inflammation or autoimmunity. The study cohort consisted of consecutive hospital admissions, without stratification or preselection based on age or sex. The characteristics of the patients are presented in Table 10.

Two 9 mL tubes of whole blood with ethylenediaminetetraacetic acid were collected. PBMCs were isolated by density gradient centrifugation using Gradisol as the reagent (Aqua-Med, Łódź, Poland). PBMCs were collected, washed with phosphate-buffered saline (PBS; EURx, Gdańsk, Poland), and subsequently suspended in 1 mL of PBS. Total RNA from the isolated cells was extracted using the DNA/RNA Extracol Kit (EURx, Gdańsk, Poland) according to the manufacturer’s instructions and stored at −80 °C for further expression analysis.

### 4.2. Quantitative Analysis of MiRNA

A total of 100 ng of RNA was reverse transcribed using the miRCURY LNA RT Kit (Qiagen, Hilden, Germany) according to the manufacturer’s instructions. The resulting cDNA samples for the quantitative PCR (qPCR) procedure were diluted eightfold. The qPCR was performed using the Fast SG qPCR Master Mix kit (EURx, Gdansk, Poland) in a QuantStudio 5 thermocycler (Thermo Fisher Scientific, Waltham, MA, USA). Thermocycling conditions followed the manufacturer’s protocol and included 45 amplification cycles with an annealing/elongation step at 60 °C for 30 s. After the qPCR procedure, melting curve analysis was performed. Primers were designed using the miRprimer2 software [38], and detailed sequences are provided in Supplementary Table S1. U6 Small Nuclear RNA (*RNU6-1*) was used as the reference gene. The ΔΔCt method was applied to analyze relative expression levels. ΔCt was calculated by subtracting the Ct value of the reference gene from the Ct value of the target gene. ΔΔCt was then determined by comparing the ΔCt of each sample to the mean ΔCt of the control group.

### 4.3. Statistics

The data’s distribution was evaluated using the Shapiro–Wilk W test. Quantitative variables with a normal distribution were presented as the mean ± SD; otherwise, the median (lower–upper quartile, IQR) was reported. Differences between two independent groups were analyzed using Student’s *t*-test or the Mann–Whitney U test. To assess differences between the control group and patients stratified by disease activity, the Kruskal–Wallis ANOVA followed by post hoc multiple comparison analysis was performed. Associations between two continuous variables were analyzed using Spearman’s rank correlation. Qualitative data are presented as counts and percentages and were evaluated using contingency tables with the χ^2^ test and Yates’ correction. ROC curve analysis was applied to evaluate the Youden index, specificity, and sensitivity of the tested parameters. To assess the diagnostic utility of the studied markers, a logistic regression model expressed as the AUC was used. Distance-weighted least-squares smoothing was applied to generate 3D plots. A *p*-value of < 0.05 was considered statistically significant. All analyses were performed using STATISTICA; version 13 (Dell Inc., Round Rock, TX, USA, 2016).

### 4.4. Interaction Between MiRNA-186 and Its Target Genes Identified Through In Silico Analysis

To investigate the potential interactions between miRNA-186 and its target genes, an in silico analysis was performed using the miRWalk tool (version 3) [39]. Only genes that were consistently identified across three databases—TargetScan, miRDB, and miRTarBase—were included in the final dataset.

## 5. Conclusions

In summary, miRNA-186 may serve as a marker of disease activity and an auxiliary diagnostic tool in rheumatoid arthritis, particularly in ACPA-negative patients. Although miRNA-186 may hold promise as a biomarker in RA, its standalone clinical utility appears limited. Given the multifactorial nature of RA and the variability in disease presentation, miRNA-186 should not be considered a substitute for established diagnostic tools. Instead, its integration with conventional markers—such as ACPAs, the ESR, and the PGA—may enhance diagnostic precision and provide a more robust framework for disease evaluation.

## Figures and Tables

**Table 1:** Differences in relative expression of microRNAs between patients with RA and healthy controls.

Micro-RNA	RA, *n* = 46	HCs, *n* = 20	*p*-Value
miRNA-186	0.26 [0.14–1.61]	0.96 [0.22–3.91]	**0.036**
miRNA-654	0.65 [0.16–1.44]	0.88 [0.15–6.23]	0.28
miRNA-425	0.44 [0.22–2.07]	0.72 [0.25–4.8]	0.19
miRNA-22	0.62 [0.26–1.39]	0.75 [0.318–3.66]	0.42
miRNA-106b	0.25 [0.1–1.6]	0.93 [0.18–4.69]	0.08

MicroRNAs expression levels were presented as relative fold changes with reference to the housekeeping gene. Data are given as the median [lower–upper quartile]. Abbreviations: HCs, healthy controls; miRNA, microRNA; RA, rheumatoid arthritis. The significant *p*-value is in bold.

**Table 2:** Differences in microRNA relative expression between RA patients divided by disease activity according to the DAS28 and healthy controls.

Micro-RNA	RA with Active Disease (DAS28 > 3.2), *n* = 30	RA with Inactive Disease (DAS28 ≤ 3.2), *n* = 16	HCs, *n* = 20	*p*-Value
miRNA-186	0.22 [0.11–1.15]	0.34 [0.22–1.7]	0.96 [0.22–3.91]	**0.04**
miRNA-654	0.34 [0.11–1.22]	0.89 [0.33–1.46]	0.88 [0.15–6.23]	0.14
miRNA-425	0.33 [0.16–1.79]	0.61 [0.37–2.73]	0.72 [0.25–4.8]	0.06
miRNA-22	0.59 [0.19–2.08]	0.75 [0.29–1.32]	0.75 [0.318–3.66]	0.66
miRNA-106b	0.18 [0.08–1.07]	0.55 [0.2–2.07]	0.93 [0.18–4.69]	0.07

MicroRNAs expression levels were presented as relative fold changes with reference to the housekeeping gene. Data are given as the median [lower–upper quartile]. Abbreviations: DAS28, disease activity score for 28 joints; HCs, healthy controls; miRNA, microRNA; RA, rheumatoid arthritis. The significant *p*-value is in bold.

**Table 3:** Differences in microRNA relative expression between patients with RA divided by disease activity according to the SDAI and healthy controls.

Micro-RNA	RA with Active Disease (SDAI > 11), *n* = 32	RA with Inactive Disease (SDAI ≤ 11), *n* = 14	HCs, *n* = 20	*p*-Value
miRNA-186	0.24 [0.11–0.96]	0.49 [0.2–1.82]	0.96 [0.22–3.91]	**0.03**
miRNA-654	0.32 [0.12–1.19]	1.05 [0.54–1.48]	0.88 [0.15–6.23]	0.054
miRNA-425	0.32 [0.17–1.51]	1.08 [0.43–2.81]	0.72 [0.25–4.8]	**0.03**
miRNA-22	0.76 [0.19–1.65]	0.57 [0.32–1.39]	0.75 [0.318–3.66]	0.69
miRNA-106b	0.18 [0.08–1.02]	0.55 [0.14–2.54]	0.93 [0.18–4.69]	0.08

MicroRNAs expression levels were presented as relative fold changes with reference to the housekeeping gene. Data are given as the median [lower–upper quartile]. Abbreviations: HCs, healthy controls; miRNA, microRNA; RA, rheumatoid arthritis; SDAI, Simplified Disease Activity Index. Significant *p*-values are in bold.

**Table 4:** Differences in microRNA relative expression between patients with RA divided by disease activity according to the CDAI and healthy controls.

MicroRNA	RA with Active Disease (CDAI > 10), *n* = 32	RA with Inactive Disease (CDAI ≤ 10), *n* = 14	HCs, *n* = 20	*p*-Value
miRNA-186	0.24 [0.11–0.96]	0.34 [0.20–1.82]	0.96 [0.22–3.91]	**0.03**
miRNA-654	0.35 [0.12–1.33]	0.89 [0.34–1.48]	0.88 [0.15–6.23]	0.15
miRNA-425	0.33 [0.17–1.64]	0.61 [0.41–2.81]	0.72 [0.25–4.8]	0.06
miRNA-22	0.76 [0.19–1.66]	0.57 [0.32–1.39]	0.75 [0.318–3.66]	0.7
miRNA-106b	0.18 [0.08–0.94]	0.77 [0.14–2.54]	0.93 [0.18–4.69]	0.06

MicroRNAs expression levels were presented as relative fold changes with reference to the housekeeping gene. Data are given as the median [lower–upper quartile]. Abbreviations: CDAI, Clinical Disease Activity Index; HCs, healthy controls; miRNA, microRNA; RA, rheumatoid arthritis. The significant *p*-value is in bold.

**Table 5:** Correlation analysis between individual miRNAs in patients with rheumatoid arthritis.

micro-RNA	RA Group				
	miRNA-186	miRNA-654	miRNA-425	miRNA-22	miRNA-106b
miRNA-186	1				
miRNA-654	0.83	1			
miRNA-425	**0.92**	**0.9**	1	1	
miRNA-22	0.77	0.75	0.78	0.76	
miRNA-106b	**0.97**	0.83	**0.91**	1	1

The strongest correlations (≥0.9) are indicated in bold. Abbreviations: miRNA; microRNA, RA, rheumatoid arthritis.

**Table 6:** Correlation analysis between individual miRNAs in the healthy control group.

microRNA	HC Group				
	miRNA-186	miRNA-654	miRNA-425	miRNA-22	miRNA-106b
miRNA-186	1				
miRNA-654	0.83	1			
miRNA-425	**0.93**	0.83	1		
miRNA-22	0.73	0.59	0.86	1	
miRNA-106b	**0.96**	0.89	**0.94**	0.72	1

The strongest correlations (≥0.9) are indicated in bold. Abbreviations: miRNA; microRNA, HC, healthy control.

**Table 7:** Diagnostic potential of serological markers and selected miRNA molecules.

MicroRNA Name	AUC	*p*-Value	Youden’s Index	Sensitivity [%]	Specificity [%]
ACPA	0.907	**<0.0001**	0.74	74	**100**
RF	0.753	**<0.0001**	0.61	61	**100**
miRNA-186	0.664	**0.03**	0.29	54	**75**
miRNA-425	0.603	0.18	0.26	61	65
miRNA-106b	0.635	0.08	0.25	**81**	45
miRNA-654	0.585	0.3	0.29	**89**	40
miRNA-22	0.563	0.41	0.15	**85**	30

Abbreviations: AUC, area under the curve; for others. Please refer to previous tables. Significant *p*-values are in bold. The top three sensitivity and specificity values are in bold.

**Table 8:** Assessment of the diagnostic value of the targets studied using a logistic regression model.

Variable	AUC
ACPA	0.907
ACPA + miRNA-186	**0.937**
ACPA + miRNA-425	0.925
ACPA + miRNA-106b	0.928
ACPA + miRNA-654	**0.941**
ACPA + miRNA-22	0.903
ACPA + all miRNAs	**0.949**

Abbreviations: ACPA, anti-citrullinated protein antibody; AUC, area under the curve; for others, please refer to previous tables. The top three AUC values are bold.

**Table 9:** Assessment of the possibility of using microRNA as markers of exacerbation of rheumatoid arthritis disease using a logistic regression model.

Variable	AUC
**Clinical variables**	
ESR	0.8156
VAS PGA	0.8604
VAS PhGA	0.9562
TJN	0.9531
SJN	0.8916
TJN + SJN + ESR	0.9739
**ESR and molecular markers**	
Combination of all miRNAs	0.7125
ESR + miRNA-186	0.8270
ESR + miRNA-425	0.8166
ESR + miRNA-106b	0.8250
ESR + miRNA-654	0.8229
ESR + miRNA-22	0.8166
ESR + all miRNAs	0.8437
**Laboratory and molecular variables and data from the patient questionnaire**	
ESR + VAS PGA	0.9468
ESR + VAS PGA + miRNA-186	0.9604
ESR + VAS PGA + all miRNAs	0.9583

Abbreviations: AUC, area under the curve; DAS28, disease activity score of 28 joints; ESR, erythrocyte sedimentation rate; miRNA, microRNA; VAS PhGA, Visual Analog Scale–Physician Global Assessment; VAS PGA, Visual Analog Scale–Patient Global Assessment; SJN, number of swollen joints; TJN, number of tender joints.

**Table 10:** Characteristics of the subjects included in this study.

Characteristics	RA, *n* = 46	HC, *n* = 20	*p*–Value
Age, years	57 [51–64]	56 [50.5–60]	0.32
Females, n (%)	40 (87)	15 (75)	0.4
Duration of the disease, years	12 [6–22]	n/a	n/a
RF-positive, n (%)	28 (60.87)	1 (5)	**<0.001**
RF (U/mL)	51.7 [12.93–157.67]	11.31 [10.09–16.34]	**0.001**
ACPA positive, n (%)	34 (73.91)	0	**<0.001**
ACPA (U/mL)	186.28 [19.19–569.07]	7.11 [5.56–10.82]	**<0.001**
ESR, mm/h	17 [8–36]	8.5 [6–13.5]	**0.018**
DAS28-ESR	4.73 [3.01–5.92]	n/a	n/a
CDAI	21.5 [9.9–32]	n/a	n/a
SDAI	22.81 [9.95–34]	n/a	n/a
Swollen joints	2 [0–6]	n/a	n/a
Tender joints	5.5 [2–14]	n/a	n/a
VAS PGA	50 [40–70]	n/a	n/a
VAS PhGA	45 [28–70]	n/a	n/a
At least csDMARD, n (%)	36 (78.3)	0	n/a
At least a biologic agent, n (%)	16 (34.8)	0	n/a
At least steroid, n (%)	35 (76.1)	0	n/a
Single-line therapy, n (%)	6 (13)	0	n/a
Double-line therapy, n (%)	30 (65.2)	0	n/a
Triple-line therapy, n (%)	7 (15.2)	0	n/a
No available information about therapy, n (%)	3 (6.5)	0	n/a

Data are presented as the median [lower–upper quartile] or number (%). Abbreviations: ACPA, anticitrullinated protein antibody; CDAI, Clinical Disease Activity Index; csDMARD, conventional synthetic disease-modifying antirheumatic drug; DAS28, disease activity score of 28 joints; ESR, erythrocyte sedimentation rate; HC, healthy control; n/a, not applicable; RA, rheumatoid arthritis; RF, rheumatoid factor; SDAI, Simplified Disease Activity Index; VAS PhGA, Visual Analog Scale–Physician Global Assessment; VAS PGA, Visual Analog Scale–Patient Global Assessments.

## Data Availability

The data generated during this study are included in the Results Section and in the Supplementary Materials.

## Supplementary Materials

The following supporting information can be downloaded at https://www.mdpi.com/article/10.3390/ijms26168039/s1.

## Abbreviations

The following abbreviations are used in this manuscript:

ACPA	Anti-Citrullinated Protein Antibodies
Ago	Argonaute protein
AMPK	AMP-activated protein kinase
AUC	Area Under the Curve
CDAI	Clinical Disease Activity Index
CDS	Coding Sequence
CRP	C-Reactive Protein
csDMARD	Conventional Synthetic Disease-Modifying Antirheumatic Drug
DAS28	Disease Activity Score for 28 Joints
ESR	Erythrocyte Sedimentation Rate
FMF	Familial Mediterranean Fever
GAS5	Growth Arrest-Specific 5 (lncRNA)
HC/HCs	Healthy Control(s)
IL-1β	Interleukin-1 beta
IL-6	Interleukin-6
IRAK1	Interleukin-1 Receptor-Associated Kinase 1
JAK	Janus Kinase
lncRNA	Long Noncoding RNA
MAPK	Mitogen-Activated Protein Kinase
miRNA	MicroRNA
ncRNA	Noncoding RNA
OA	Osteoarthritis
PBMCs	Peripheral Blood Mononuclear Cells
PCR	Polymerase Chain Reaction
PhGA	Physician Global Assessment
PGA	Patient Global Assessment
pre-miRNA	Precursor MicroRNA
pri-miRNA	Primary MicroRNA
RA	Rheumatoid Arthritis
RF	Rheumatoid Factor
RISC	RNA-Induced Silencing Complex
ROC	Receiver Operating Characteristic
SJN	Swollen Joint Number
SDAI	Simplified Disease Activity Index
SLE	Systemic Lupus Erythematosus
TJN	Tender Joint Number
TNF	Tumor Necrosis Factor
TRAF6	TNF Receptor-Associated Factor 6
UTR	Untranslated Region
VAS PhGA	Visual Analog Scale–Physician Global Assessment
VAS PGA	Visual Analog Scale–Patient Global Assessment
