# Supplementary material for: MiRNA-186 as a Biomarker of Disease Exacerbation in Rheumatoid Arthritis: Insights from Clinical Data and Molecular Marker Analysis
Source: Int J Mol Sci. 2025 Aug 20;26(16):8039. doi: 10.3390/ijms26168039 (PMC12386512; doi:10.3390/ijms26168039)
Supplement: Supplementary file 1 [file ijms-26-08039-s001.zip › ijms-3804253-supplementary.pdf]

miRNA-186 as a biomarker of disease exacerbation in rheumatoid arthritis: Insights from Clinical Data and Molecular Marker Analysis.

Ciesla M. et al.

Table S1. Primer sequences used for the polymerase chain reaction.

Reference gene name	Accession number	Primer sequence	Product sequence<br>5' to 3'
RNU6-1 (U6 Small Nuclear 1 gene)	M14486	Forward: 5'CTCGCTTCGGCAGCACA	GTGCTCGCTTCGGCAGCACATATACTAAAATTG GAACGATACAGAGAAGATTAGCATGGCCCCTG CGCAAGGATGACACGCAAATTCGTGAAGCGTT CCATATTTTG
		Reverse: 5'AACGCTTCACGAATTTGCGT	
**miRNA Name**	**Accession number**	**Primer sequence**	**Product Sequence<br>5' to 3'**
miRNA 186-5p	MIMAT0000456	Forward: 5' ATCGCAGCAAAGAATTCTCCT	CAAAGAAUUCUCCUUUUGGGCU
		Reverse: 5'AGTTTTTTTTTTTTTTTAGCCCAA	
miRNA 654-3p	MIMAT0004814	Forward:5'ACATCAGTATGTCTGCTGACCATC	UAUGUCUGCUGACCAUCACCUU
		Reverse: 5'CCAGTTTTTTTTTTTTTTTAAGGTG	
miRNA 425-5p	MIMAT0003393	Forward: 5' GGCAGAATGACACGATCACTCC	AAUGACACGAUCACUCCCGUUGA
		Reverse: 5'GGTCCAGTTTTTTTTTTTTTTTCAAC	
miRNA 22-3p	MIMAT0000077	Forward: 5'CGAGAAGCTGCCAGTTGAAGA	AAGCUGCCAGUUGAAGAACUGU
		Reverse: 5' GGTCCAGTTTTTTTTTTTTTTTACAGT	
miRNA 106b-5p	MIMAT0000680	Forward: 5'CTAATGGCAAAGTGCTCATAGTG	UAAAGUGCUGACAGUGCAGAU
		Reverse: 5'GGTCCAGTTTTTTTTTTTTTTTCTAC	

Table S2. MicroRNA expression in patients divided according to the presence of rheumatoid factor and in healthy individuals.

Micro-RNA	RA with positive RF, n = 28	RA with negative RF, n=18	HC, n=20	p-value
miRNA-186	0.31 [0.21 – 1.5]	0.16 [0.11 – 1.82]	0.96 [0.22 – 3.91]	0.07
miRNA-654	0.7 [0.18 – 1.46]	0.34 [0.15 – 1.22]	0.88 [0.15 – 6.23]	0.52
miRNA-425	0.46 [0.27 – 1.85]	0.33 [0.17 – 2.07]	0.72 [0.25 – 4.8]	0.36
miRNA-22	0.75 [0.24 – 1.73]	0.59 [0.38 – 1.21]	0.75 [0.318 – 3.66]	0.72
miRNA-106b	0.45 [0.17 – 1.29]	0.13 [0.08 – 1.6]	0.93 [0.18 – 4.69]	0.15

Data are presented as median [lower – upper quartile]. Abbreviations: HC, healthy control group; RA, patients with rheumatoid arthritis; RF, rheumatoid factor.

Table S3. MicroRNA expression in patients divided according to the presence of anticitrullinated protein antibodies and in healthy individuals.

MicroRNA	RA with ACPA positive, n=34	RA with ACPA negative, n=12	HC, n=20	p-value
miRNA-186	0.29 [0.16 – 1.89]	0.16[0.12 – 0.73]	0.96 [0.22 – 3.91]	0.04
miRNA-654	0.65 [0.19 – 2.43]	0.59 [0.14 – 1.03]	0.88 [0.15 – 6.23]	0.42
miRNA-425	0.46 [0.26 – 2.39]	0.29 [0.17 – 1.12]	0.72 [0.25 – 4.8]	0.19
miRNA-22	0.8[0.26 – 2.38]	0.53 [0.28 – 0.78]	0.75 [0.318 – 3.66]	0.44
miRNA-106b	0.37 [0.09 – 1.64]	0.16 [0.1 – 0.89]	0.93 [0.18 – 4.69]	0.17

Data are presented as median [lower – upper quartile]. Abbreviations: ACPA, anticitrullinated protein antibodies; HC, healthy control group; RA, patients with rheumatoid arthritis.

Table S4. Spearman's rank correlation between clinical variables and microRNAs expression in patients with rheumatoid arthritis assessed by quantitative PCR.

	MicroRNA				
**Clinical Variable**	miRNA-186	miRNA-654	miRNA-425	miRNA-22	miRNA-106
DAS28	-0.075184	-0.254364	-0.218337	-0.094304	-0.084004
SDAI	0.001383	-0.087492	-0.106858	0.026945	0.008367
CDAI	0.005923	-0.047416	-0.094768	0.064289	0.008514
TJN	0.067668	-0.047295	-0.049929	0.014017	0.047262
SJN	-0.019996	-0.155336	-0.158823	-0.050338	-0.007300
ESR	-0.133885	**-0.294921**	-0.256037	-0.186439	-0.163219
RF [U/ml]	0.156460	0.027630	0.061980	-0.003269	0.127475
ACPA [IU/ml	0.200617	0.065620	0.180142	0.131915	0.154487

Abbreviations: *ACPA, anticitrullinated protein antibodies; CDAI, Clinical Disease Activity Index; DAS28, disease activity score of 28 joints; ESR, erythrocyte sedimentation rate; RF, rheumatoid factor; SDAI, Simplified Disease Activity Index; SJN, swollen joints number; TJN, tender joints number; Significant correlation was in bold and red indicated.*

Table S5. Interactions between miRNA-186 and its predicted target genes based on in silico analysis.

CDS							
**MiRNA id**	**Refseq id**	**Gene symbol**	**Binding site**	**Score**	**Au**	**Me**	**Number of pairings**
hsa-miR-186-5p	NM_001365684	NSD1	3782, 3798	1	0.456	-17.110	15
	NM_001365684	NSD1	4431, 4468	1	0.471	-4.957	19
	NM_001371910	MAP3K2	1120, 1166	1	0.559	-9.547	17
	NM_001371911	MAP3K2	814, 860	1	0.559	-9.547	17
	NM_017934	PHIP	5329, 5346	1	0.603	-16.809	15
	NM_018221	MOB1A	345, 365	1	0.544	-9.547	17
	NM_022455	NSD1	4685, 4701	1	0.456	-17.110	15
	NM_001167	XIAP	1591, 1623	1	0.574	-7.3822	19
	NM_002158	FOXN2	505, 527	1	0.588	-6.858	19
	NM_002264	KPNA1	259, 273	1	0.485	-19.424	12
	XM_005248729	PHIP	5326, 5343	1	0.603	-16.809	15
**3'UTR**							
**MiRNA id**	**Refseq id**	**Gene symbol**	**Binding site**	**Score**	**Au**	**Me**	**Number of pairings**
hsa-miR-186-5p	NM_173511	FAM117B	2355, 2368	1	0.529	-19.424	12
	NM_024528	NKAP	5714, 5734	1	0.603	-10.075	17
	NM_001005849	SUMO2	747, 766	1	0.75	-8.1068	16
**5'UTR**							
**MiRNA id**	**Refseq id**	**Gene symbol**	**Binding site**	**Score**	**Au**	**Me**	**Number of pairings**
hsa-miR-186-5p	XM_005263236	NAA15	263, 315	1	0.441	-5.926	17

Abbreviations: *Au, adenine and uracil-rich region; CDS, coding sequence; Me, m/e motif; miRNA, micro-RNA; UTR, untranslated region.*
